# Enhancing Ocular Bioavailability of Ciprofloxacin Using Colloidal Lipid-Based Carrier for the Management of Post-Surgical Infection

**DOI:** 10.3390/molecules27030733

**Published:** 2022-01-23

**Authors:** Fakhria A. Al-Joufi, Mounir M. Salem-Bekhit, Ehab I. Taha, Mohamed A. Ibrahim, Magdy M. Muharram, Sultan Alshehri, Mohammed M. Ghoneim, Faiyaz Shakeel

**Affiliations:** 1 Department of Pharmacology, College of Pharmacy, Jouf University, Al-Jouf 72341, Saudi Arabia; faaljoufi@ju.edu.sa; 2 Department of Pharmaceutics, College of Pharmacy, King Saud University, Riyadh 11451, Saudi Arabia; eelbadawi@ksu.edu.sa (E.I.T.); mhamoudah@ksu.edu.sa (M.A.I.); salshehri1@ksu.edu.sa (S.A.); fsahmad@ksu.edu.sa (F.S.); 3 Microbiology and Immunology Department, Faculty of Pharmacy, Al-Azhar University, Cairo 11651, Egypt; 4 Department of Pharmaceutics, College of Pharmacy, Prince Sattam Bin Abdulaziz University, Al-Kharj 11942, Saudi Arabia; m.moharm@psau.edu.sa; 5 Department of Microbiology, College of Science, Al-Azhar University, Nasr City, Cairo 11884, Egypt; 6 Department of Pharmacy Practice, College of Pharmacy, AlMaarefa University, Ad Diriyah 13713, Saudi Arabia; mghoneim@mcst.edu.sa

**Keywords:** bioavailability, ciprofloxacin, lipid-based colloidal carriers, ocular delivery

## Abstract

Conjunctivitis and endogenous bacterial endophthalmitis mostly occurred after ophthalmic surgery. Therefore, the present study aimed to maximize the ocular delivery of ciprofloxacin (CPX) using colloidal lipid-based carrier to control the post-surgical infection. In this study, CPX was formulated as ophthalmic liposomal drops. Two different phospholipids in different ratios were utilized, including phosphatidylcholine (PC) and dimyrestoyl phosphatidylcholine (DMPC). The physiochemical properties of the prepared ophthalmic liposomes were evaluated in terms of particle size, entrapment efficiency, polydispersity index, zeta potential, and cumulative CPX in-vitro release. In addition, the effect of sonication time on particle size and entrapment efficiency of CPX ophthalmic drops was also evaluated. The results revealed that most of the prepared formulations showed particle size in nanometer size range (460–1047 nm) and entrapment efficiency ranging from 36.4–44.7%. The antibacterial activity and minimum inhibitory concentration (MIC) were investigated. Ex vivo antimicrobial effect of promising formulations was carried out against the most common causes of endophthalmitis microorganisms. The pharmacokinetics of the prepared ophthalmic drops were tested in rabbit aqueous humor and compared with commercial CPX ophthalmic drops (Ciloxan^®^). Observed bacterial suppression was detected in rabbit’s eyes conjunctivitis with an optimized formulation A3 compared with the commercial ophthalmic drops. CPX concentration in the aqueous humor was above MIC against tested bacterial strains. The in vivo data revealed that the tested CPX drops showed superiority over the commercial ones with respect to peak aqueous humor concentration, time to reach peak aqueous humor concentration, elimination rate constant, half-life, and relative bioavailability. Based on these results, it was concluded that the prepared ophthalmic formulations significantly enhanced CPX bioavailability compared with the commercial one.

## 1. Introduction

Ciprofloxacin (CPX) is one of the most effective antibiotics, active against the vast range of infection causing ophthalmic pathogens. It blocks the bacterial deoxyribonucleic acid (DNA) synthesis via inhibition of DNA gyrase [1,2]. CPX lipophilicity is high enough to permeate via ocular humors and it is the preferred therapy for intraocular infections [3]. When CPX is dispensed locally into the conjunctival sac at specific concentration, it infiltrates into the aqueous humor and its concentration is reliant on the dose number [4]. Therefore, it has been verified to be an effective local antimicrobial for the application as a sole drug for conjunctivitis and keratitis treatment [5]. CPX has also been proposed for prophylactic use in cases of endogenous bacterial endophthalmitis. Different studies reported the most common causes of endophthalmitis include *Staphylococcus aureus*, *Bacillus* spp., *Escherichia coli*, *Pseudomonas* spp., and *Klebsiella* spp. [6]. CPX interacts with infectious bacteria at the site of infection, not in the blood stream. Therefore, in order to prevent bacterial growth, CPX must be delivered at a high concentration to the infected area [7].

For marketed CPX ophthalmic drops to be effective, frequent dosing to eye sac must be applied because all marketed CPX ophthalmic drops utilize aqueous solutions [8,9]. The solubility of CPX is optimum in acidic pH (around 4.5); however, the pH of the tears is in the neutral range (almost 7) [10]. This way of administering the commercial CPX ophthalmic drops is always accompanied by burning sensation and itching [11]. In addition, because of the negative charge in the corneal surface (due to presence of a thin layer of negatively charged mucin), a positively charged carrier would result in increasing the drug resident time in the eye [12]. Stearylamine (STA) is a well-known positive charge-inducing agent. Thus, it was utilized in the formulations to increase the contact time, prolong the drug release, and improve CPX bioavailability.

Liposomes are vesicular systems (usually within the size range of 10 nm to 1 μm or greater) composed of an aqueous core enclosed by phospholipid bilayers of natural or synthetic origin. Liposomes are advantageous in encapsulating both lipophilic and hydrophilic molecules. Hydrophilic drugs are entrapped in the aqueous layer, while hydrophobic drugs are stuck in the lipid bilayers [13]. Liposomes have been considered for ocular administration because they pose ophthalmic drug delivery advantages. They are biocompatible and biodegradable nanocarriers and can enhance the permeation of poorly absorbed drug molecules by binding to the corneal surface and prolonging residence time [14].

This study aims to design CPX loaded colloidal lipid-based carriers (ophthalmic liposomal drops) to enhance its ocular bioavailability in order to reduce and control surgical site infections after ophthalmic operation. The pharmacokinetics parameters such as peak aqueous humor concentration (C_max_), time to reach peak aqueous humor concentration (T_max_), half-life (t_1/2_), and area under curve from time zero to t (AUC_0–t_) were used to evaluate the prepared CPX drops in comparison with the marketed one in the rabbit model. In order to achieve this goal, several vesicular formulations utilizing phospholipids such as phosphatidylcholine (PC) and dimyrestoyl phosphatidylcholine (DMPC) were used. The prepared CPX ophthalmic drops were evaluated in terms of particle size, entrapment efficiency, polydispersity index, zeta potential, and in vitro CPX release rate.

## 2. Results and Discussion

### 2.1. Polydispersity Index (PDI) and Zeta Potential of CPX Colloidal Lipid-Based Formulations (CPX-CLBFs) 

The PDI and zeta potential values of different CPX-CLBFs formulations were determined as described in the experimental section. The PDIs of different formulations were found to be 0.128–0.767. The minimum PDI was recorded for formulation B3 (0.128), indicating the maximum uniformity in particle size distribution compared to other liposomal formulations evaluated. The maximum PDI was recorded for formulation A0 (0.767), indicating broad size distribution in formulation A0. The zeta potential of different formulations was obtained as 3.6–25.7 mV. The minimum and maximum zeta potential values were recorded for formulations A0 (3.6 mV) and A3 (25.7 mV), respectively. The zeta potential value in the range of ±30 mV indicated the maximum stability of formulations [15]. The zeta potential of liposomal formulation A3 was found to be much closed with ±30 mV, indicating the maximum stability of liposomal formulation A3 compared to the other formulations studied. 

### 2.2. Influence of Phospholipids Type on Ophthalmic CPX-CLBFs

**Figure 1:**
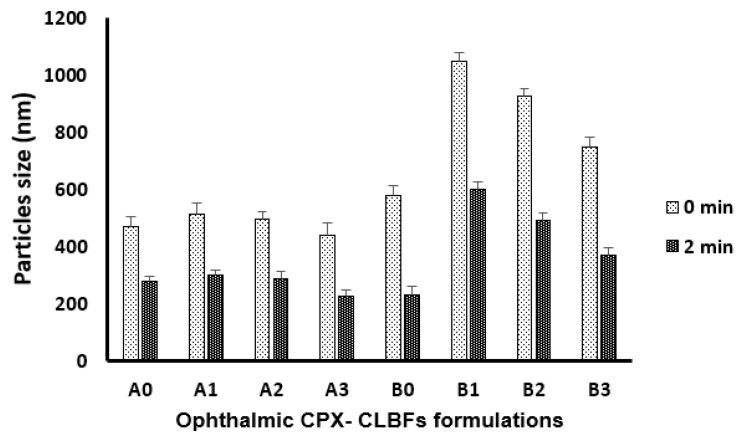
Effect of sonication time on particle size of ophthalmic ciprofloxacin (CPX) colloidal lipid-based formulations (CPX-CLBFs) (mean ± SD, *n* = 3.0).

**Figure 2:**
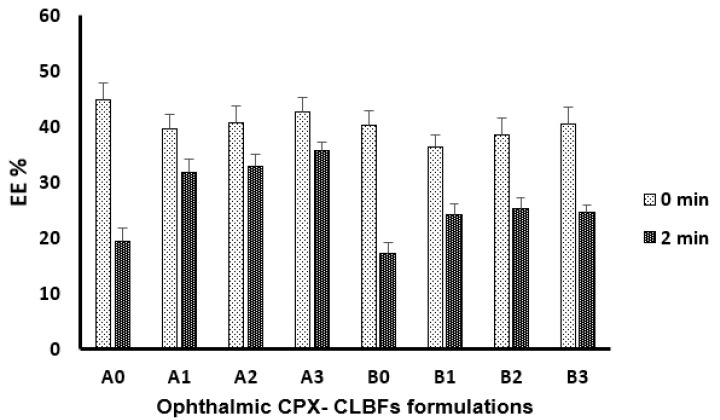
Effect of sonication time on percent entrapment efficiency (EE%) of ophthalmic CPX-CLBFs (mean ± SD, *n* = 3.0).

Figure 1 and Figure 2 show the effect of phospholipids type on particle size and the percent of entrapment efficiency (EE%) of ophthalmic CPX-CLBFs. It was obvious that the presence of DMPC phospholipid (formulations B0 and B1–B3) in large amounts produced larger particle sizes compared with the incorporation of PC in the formulations (A0 and A1–A3). Among the different PC formulations studied (A0 and A1–A3), the particle size of formulation A3 was smallest compared with formulations A0, A1, and A2. These observations indicated that the amount of PC had a definite impact on particle size of PC formulations. The particle size of PC formulations was found to be decreased with increase in the concentration of PC in the formulations (Figure 1). Among different DMPC formulations studied (B0 and B1–B3), the particle size of formulation B0 was smallest compared with formulations B1–B3. These observations also indicated that the amount of DMPC had a definite impact on the particle size of DMPC formulations. The particle size of DMPC formulations was also found to be decreased with increase in the concentration of DMPC in the formulations (Figure 1). On the other hand, phospholipids type had little effect on EE% of the prepared ophthalmic CPX-CLBFs, which could be due to the 36 carbon atoms for the hydrophobic part of PC molecules compared with 28 carbon atoms for hydrophobic part of DMPC. Among different PC formulations studied (A0 and A1–A3), the EE% of formulation A0 was highest compared with formulations A1–A3. However, the EE% of different PC formulations was not significantly different. These observations indicated that the amount of PC had little impact on EE% of PC formulations (Figure 2). Among different DMPC formulations studied (B0 and B1–B3), the EE% of formulation B3 was highest compared with other DMPC formulations studied. However, the EE% of different DMPC formulations was not significantly different. These observations also indicated that the amount of DMPC had little impact on EE% of DMPC formulations (Figure 2). Gulati et al. observed that drug entrapment was increased by increasing the carbon chain length of the phospholipids used [16]. Our results were in good agreement with those reported by Gulati et al. [16].

### 2.3. Impact of Sonication on Ophthalmic CPX-CLBFs

Figure 1 and Figure 2 show the impact of sonication time on ophthalmic CPX-CLBFs particle size and EE%. It was clear that 2 min of sonication time decreases the particle size of all formulations. In addition, it was observed that CPX-CLBFs containing cholesterol (CL) are not able to increase EE% due to high membrane rigidity of CL. As a result, the values of EE% for formulations containing CL (A1–A3 and B1–B3) was decreased after sonication for 2 min (Figure 2) due to the presence of CL which minimized CPX leakage from CLBFs upon sonication. 

### 2.4. In Vitro Release Study from Ophthalmic CPX-CLBFs

**Figure 3:**
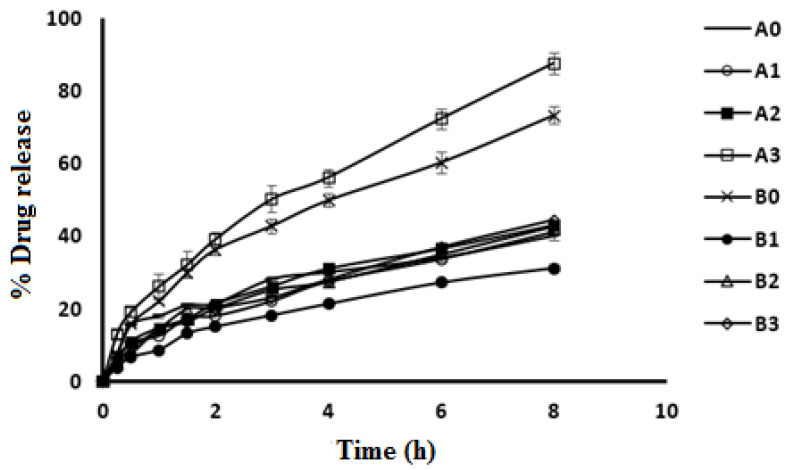
In vitro release profile of CPX from different ophthalmic CPX-CLBFs (mean ± SD, *n* = 3.0).

Figure 3 shows the in vitro release profile of CPX form ophthalmic CPX-CLBFs. All formulations showed small initial gradual CPX release. Both B0 and A3 showed a faster release compared with other formulations with cumulative CPX percent release of 73% and 87%, respectively. CPX release was found to depend mainly on formulations particle size. Formulations with small particle size showed high cumulative drug percent release. 

### 2.5. Antibacterial Activity of Different CPX-CLBFs Formulations

The antibacterial activity and the minimum inhibitory concentration (MIC) were evaluated using the cup plate method and microdilution method, respectively, against bacterial strains frequently responsible for endophthalmitis. The MIC values of liposomal formulation samples containing CPX were investigated and the most effective are presented in Table 1. All formulations showed weak antibacterial activity (MIC ranged from 64–128 μg/mL) against all the tested strains except for formulations A3 and B0, which displayed a potent antibacterial activity (MIC ranged from 16–32 μg/mL) against all the tested strains. These formulations showed obvious activities in comparison with the other formulations against Gram-positive and -negative strains. For the *S. aureus*, *B. subtilis*, and *P. aeruginosa* bacterial strains, A3 revealed the best strength with MIC values of 16 μg/mL and exhibited minimum bactericidal concentration (MBC) values lower than that of the reference CPX. The higher in vitro antibacterial activity of formulations A3 and B0 were possibly due to the faster release of CPX from formulations A3 and B0 compared with other formulations studied.

The microbiological susceptibility testing of released CPX from the lipid-based entrapped CPX formulations was tested against different strains of Gram-negative (*E. coli*, *P. aeruginosa*, and *K. pneumonia*) and Gram-positive (*S. aureus*, *E. faecalis*, and *B. subtilis*) bacteria. These organisms are implicated in endophthalmitis [17]. Donnenfeld et al. alleged that the most common bacteria accountable for endophthalmitis were *S. aureus*, *S. epidermidis*, and *Streptococcus* sp. (50%, 30%, and 10%, respectively) [18]. The mean concentrations of antibiotic achieved in the current study were less than 1 μg/mL. The reported MIC of CPX required to inhibit the growth of *S. aureus* and *P. aeruginosa* ranged from 0.125–1 µg/mL [19]. This result was in agreement with Cutarelli et al. who reported a MIC90 of 1 μg/mL for CPX against tested strains [20]. Lesk et al. also estimated as low as 0.4 μg/mL for *S. epidermidis* and up to 1 μg/mL for *B. cereus* [21]. Therefore, the most effective two lipid-based entrapped CPX formulations were selected for the in vivo evaluation (B0 and A3). Both CPX-CLBF and Ciloxan^®^ eye drop improved MIC and MBC of *P. aeruginosa* and *S. aureus*. Jain and Shastri reported a comparable result of liposomal formulation on MIC and MBC of CPX [22].

**Figure 4:**
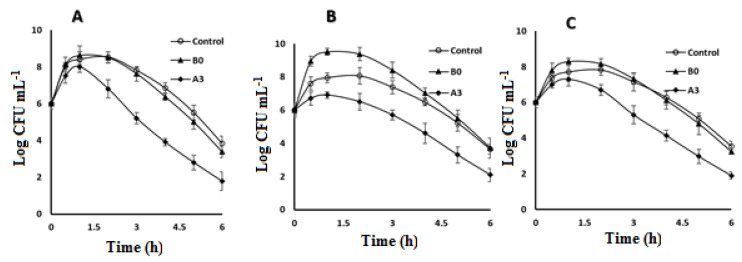
Colony count of the infected aqueous humor after the topical application of B0 and A3 formulations and Ciloxan^®^ eye drops against *S. aureus* (**A**), *K. pneumonia* (**B**), and *P. aeruginosa* (**C**), (mean ± SD, *n* = 3.0).

The results of the antimicrobial effectiveness and the level of CPX in the conjunctival sac of tested rabbits following the topical application of B0 and A3 formulations and Ciloxan^®^ eye drops are shown in Figure 4. The reduction in bacterial count was detected and better improvement was observed in rabbit’s eyes conjunctivitis. The ex vivo results were consistent with the results of the in vitro release, where the lipid-based entrapped CPX formulations A3 showed the uppermost CPX levels in the conjunctival sac in comparison to retailed Ciloxan^®^ drops. B0, when compared to A3 and commercial eye drops, significantly failed to reduce the bacterial growth, which could be due to lack of positively charging agent. Effectually, cationic molecules such as STA are deemed to be notable bio-adhesives owing to an ability to produce forces of molecular attraction through the electrostatic interactions with the negative charges of mucin coating corneal surface as well as the secretions at the site of infection.

### 2.6. Pharmacokinetic Study

**Figure 5:**
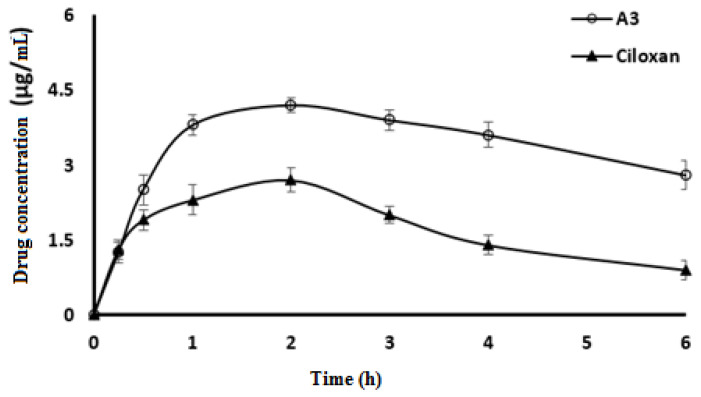
CPX concentrations in rabbit aqueous humor for ophthalmic CPX-CLBF (A3) compared with commercial one (mean ± SD, *n* = 6.0).

Figure 5 shows CPX concentration in aqueous humor following application of CPX-CLBF A3 compared with Ciloxan^®^. Table 2 shows the pharmacokinetic parameters of A3 and Ciloxan^®^. One–way ANOVA was utilized to analyze the calculated pharmacokinetic parameters after application of CPX-CLBF A3 and Ciloxan^®^. The C_max_ of 4.2 μg/mL was obtained from A3 compared with 2.7 μg/mL for Ciloxan^®^. There were no significant differences in values of T_max_ between A3 and Ciloxan^®^. The values of AUC_0-t_ for A3 (34.9 μg min/mL) were significantly greater than those obtained from Ciloxan^®^ (12 μg min/mL). These results confirmed a greater ocular bioavailability of CPX-CLBF A3. High values of AUC_0–t_ indicated high extent of drug absorption. Regarding t_1/2_, A3 shows increased t_1/2_ value compared with that of the commercial formulation which indicated the presence of CPX for longer time in aqueous humor. The relative bioavailability valued significantly emphasize that A3 increased drug bioavailability by 2.9-folds compared with the commercial formulation. Based on the bioavailability results, the CLBF delivered extra CPX into the aqueous humor and enhanced therapeutic effects in comparison with Ciloxan^®^ drop.

## 3. Materials and Methods

### 3.1. Materials

CPX was obtained from Fluka Biochemika (Busch, Switzerland). PC, CL, DMPC, and STA were obtained from Avanti Polar Lipids Inc. (Alabaster, AL, USA). Chloroform was obtained from BDH Laboratory Ltd. (Poole, England, UK). The solvents used in this study were of chromatography grade.

### 3.2. Bacterial Culturing

The growing bacterial cultures were determined by controlling the turbidity changes with the measurements of absorbance at 600 nm. Applying a titration curve, the changes in the absorbance were then turned correspondingly into CFU/mL (colony forming units). The titration curve was created by considering the bacterial samples with optical density of 0.05–2.0 at 600 nm. Serial dilution for the cells was performed in trypticase soy broth (Oxoid, Lenexa, KS, USA) and then culturing the diluted cells on Mueller–Hinton agar (MHA) (Oxoid, Lenexa, KS, USA). The plates were incubated for overnight at 35 ± 1 °C. Bacterial colonies were calculated, and result obtained from triplicate assays were applied to generate a titration curve that compared optical density measurements with CFU/mL for the bacterial strains growth.

### 3.3. Preparation of CPX-CLBFs

CPX-CLBFs were prepared utilizing reverse evaporation technique [23]. The molar ratios of CPX-CLBFs are shown in Table 3. The exact amount of lipids was dissolved in 13 mL of chloroform. Five mL of CPX was dissolved in phosphate buffer (pH 4.5) and added to the mixture. Chloroform was evaporated from the mixture by rotary evaporation at 40 °C. The mixture was centrifuged for 40 min at 30,000 rpm. Free CPX content in supernatant was detected and the lipid capsules were dispersed in phosphate buffer (pH 7.4) [24]. Each formulation was subjected to sonication for 2 min right after preparation.

### 3.4. Characterization of CPX-CLBFs

#### 3.4.1. Particle Size, PDI, and Zeta Potential Measurements 

The mean particle size and PDI for each CPX-CLBFs were determined at ambient temperature using laser diffraction analysis (NiComp Particle Size system ZW380 Santa Barbara, CA, USA). Each CPX-CLBF formulation was diluted with phosphate buffer before analysis.

#### 3.4.2. Evaluation of CPX EE%

Each CPX-CLBF formulation was centrifuged for 40 min at 30,000 rpm to get rid of free CPX content and EE% was calculated using the following equation [25,26]:

(1) $$\mathrm{EE}\%=\frac{\left(W_{t}-W_{f}\right)}{W_{t}}\times 100$$

where, W_t_ and W_f_ are the total CPX and the free CPX content, respectively.

#### 3.4.3. Effect of Sonication on CPX-CLBFs Particle Size and EE%

To study the impact of sonication on particle size and EE% of CLBFs, all formulations were sonicated for 2 min. The data were recorded before and after sonication.

### 3.5. CPX Assay Method

Reversed-phase high performance liquid chromatographic (HPLC) method was used for the quantification of CPX. A mobile phase was a mixture of acetonitrile: methanol: 0.05 acetate buffer (10:20:70, *v*/*v*/*v*) and 1% (*v*/*v*) of acetic acid. The acetate buffer was at pH 3.6. The mobile phase was degassed using sonication and filtered through 0.45 µm Millipore filter immediately after preparation. Samples of 20 μL were injected in Shimadzu HPLC system consists of Fluorescence detector, pump, Kromasil 100, C18, 5 µm (250 × 4.6 mm) column, and Rheodyne sample injector were used in the assay method. CPX and anthranilic acid (internal standard) was detected at λ_exc_ = 300 nm and λ_emi_ = 458 nm, respectively, using mobile flow rate of 0.8 mL/min at room temperature [27].

### 3.6. In Vitro CPX Release Study from CLBFs

Franz diffusion cells were used to carry out this experiment. The in vitro release of CPX was performed in release medium of artificial tears (pH 7.4) placed in receptor cells [28]. A semipermeable membrane with a molecular weight cut-off range of 12,000–14,000 Da was used [29]. The membrane was soaked in the dissolution medium for about 12 h before conducting the experiment. The utilized membrane was able to permeate CPX molecules and hold CLBF vesicles. The temperature was kept at 34 ± 0.5 °C and the mixture was stirred at 300 rpm. A specific amount of each CPX ophthalmic formulation was placed in the dialysis donor cell. Samples of 1 mL each were periodically collected at time intervals of 0, 0.25, 0.5, 1, 2, 3, 4, and 6 h and replaced by same volume of artificial tears to maintain constant volume and sink condition. Then, the amount of CPX was determined in each sample using HPLC method described above [27]. All tests were repeated in triplicate. Based on the characterizations of ophthalmic CPX-CLBFs formulations, A3 (after sonication) and B0 (without sonication) were selected for further investigation.

### 3.7. Evaluation of the In Vitro Antibacterial Activity of CPX-CLBFs

Antibiogram was carried out for the prepared 8 colloidal lipid-based entrapped CPX formulations, as well as the standard CPX-hydrochloride, by the agar well diffusion method [30,31]. CLBF vesicles were separately investigated against a set of Gram-positive and -negative bacterial strains as proposed by the Clinical Laboratory Standard Institute (CLSI) guidelines [32]. For this purpose, different strains of Gram-negative bacteria (*E. coli* ATCC 9637, *P. aeruginosa* ATCC 27953, and *K. pneumonia* ATCC 10031) as well as three different strains of Gram-positive bacteria (*S. aureus* ATCC 29213, *E. faecalis* ATCC 29212, and *B. subtilis* ATCC 10400) were selected. Bacterial suspension (100 μL) comprising 1 × 10^6^ CFU/mL of bacterial strain were mixed with MHA medium. After waiting for the media to solidified, wells of 6.0 mm diameter were made through the solidified MHA using a cork borer and burdened equal quantities of each tested liposomal formulation samples. The implanted plates were then overnight incubated at 35 ± 1 °C. Negative control was prepared using free MHA. The zone of inhibition against the tested organisms was measured after incubation time for evaluating the antimicrobial activity. Each test was performed in triplicate and inhibition zone average was calculated. The average diameters of the inhibition zones were recorded and expressed according to the following score guide in mm as follows: + = ≥ 6 mm; ++ = ≥ 10 mm (moderate); +++ = ≥ 15 mm (strong); and ++++ = ≥20 mm (very strong).

### 3.8. Determination of In Vitro MIC and MBC

The MIC of the most effective CPX-CLBF formulations (3 efficient formulae) and commercial ophthalmic formulation containing 0.3% CPX-hydrochloride (Ciloxan^®^ drop, Alcon Laboratories Inc., Fort Worth, TX, USA) were assayed against mentioned bacteria that are frequently responsible for endophthalmitis [33]. The test was performed by broth microdilution method [34] in conformity with the European Committee on Antimicrobial Susceptibility Testing (EUCAST) [35]. Overnight bacterial suspensions were adjusted to 1 × 10^6^ CFU/mL as described above. The CLBF samples and CPX commercial formula were adjusted to give appropriate concentration by sequential dilutions in 2X MH broth (Merck, Darmstadt, Germany). Equal bacterial inoculums (5 μL) were dispensed into wells and the microtiter plate was incubated overnight at 35 ± 1 °C. Subsequently, the MIC results were listed and explicated consistent with the EUCAST guidelines. Positive control (inoculum and MH media, devoid of liposomal entrapped CPX) was used. Free culture medium was used as a negative control. To ensure reproducibility, each assessment was completed in triplicates on three different days. Wells displaying no bacterial growth were streaked on MH agar and incubated overnight for MBC. Tested MBC was considered as the lowest concentration of either free or liposomal CPX that able to cause reduction (more than 99.9%) of the initial inoculum. 

### 3.9. Ex Vivo Antibacterial Effect of CPX-CLBFs and Ciloxan^®^

Male New Zealand white Albino rabbits (weighed 2–2.3 kg) were obtained from the Experimental Animal Care Center (EACC) at Prince Sattam bin Abdulaziz University, Al-Kharj, Saudi Arabia and utilized to conduct the in vivo/ex vivo antibacterial study. All animals were provided free access to water but were starved for 24 h before the experiment. The in vivo antibacterial studies were approved by the Animal Ethics Committee of the EACC Board (Prince Sattam bin Abdulaziz University, Al-Kharj, Saudi Arabia with approval number: BERC-005-04-21). The animal use and experimental procedures followed EU directive 2010/63/EU.

The bacterial suspensions were prepared by overnight growing at 35 ± 1 °C in trypticase soy broth and adjusted to achieve a concentration of 1 × 10^3^ CFU/30 μL, proper for ocular application. Rabbits were randomly selected and divided into three equal groups for each bacterial strain: group A for *S. aureus*, group B for *K. pneumonia*, and group C for *P. aeruginosa*. Each group contained 9 rabbits with the same race, gender and average weight of 2.0–2.3 kg. To induce intraocular infections in the rabbit eyes, 5 μL of each bacterial suspension was inoculated into the conjunctival sac through sterile dropper for each group. After infection verification, the right eyes were treated by CLBF and Ciloxan^®^ drops for the assessment of the in vivo antibacterial efficacy of different CPX-CLBF formulations. On the other hand, the left eyes were kept un-treated. From anterior chamber paracentesis, the aqueous humor specimens were withdrawn via the translimbal pathway by a 27-gauge needle mounted on a tuberculin syringe at a dose of 0.1 mL, at zero (before pouring the eye drops), 0.5, 1, 2, 3, 4, 5, and 6 h after application of the formulated drug. The CPX-CLBF in aqueous humor specimens were measured by culturing on TSA media for the microbial count and estimation of MIC and MBC. Antibacterial activities were expressed as inhibition diameter zones in mm. MIC was expressed in μg/mL and evaluated by microbroth dilution method according to EUCAST.

### 3.10. Ex Vivo Antibacterial Effect of Selected CPX-CLBFs and Ciloxan^®^ Drop

Both MIC and MBC of the prepared CLBF formulations in ex vivo media (media enriched by infected aqueous humor specimens) against *S. aureus*, *K. pneumonia*, and *P. aeruginosa* were examined (data not presented). CPX entrapped in A3 formulation decreased MIC and MBC against *K. pneumonia* and *S. aurous* more than B0 and Ciloxan^®^. A3 significantly enhanced the susceptibility of *K. pneumonia* and *S. aurous* to CPX compared with B0 and Ciloxan^®^. On the other hand, only A3 decreased MIC and MBS for *P. aeruginosa* and no significant difference was noticed between B0 and Ciloxan^®^.

### 3.11. Study of CPX-CLBFs Treatment Effect 

For the evaluation of the treatment outcome, colony count was determined in treated and un-treated rabbit’s eyes. Selected rabbits infected by *S. aureus*, *K. pneumonia*, and *P. aeruginosa* were treated by B0, A3, and Ciloxan^®^. After 6 h of treatment, the samples withdrawn from the infected aqueous humor and streaked for colony count. According to these results, A3 displayed significantly reduced colony counts for all tested organisms after 6 h. Although Ciloxan^®^ showed reducing effect in colony count, but it is insignificant compared to A3 effect. Blank CLBF control did not show any effect on demonstrated bacterial growth. On the other hand, bacterial count was significantly higher than that observed with A3 and commercial ophthalmic drops, which means B0 failed to reduce the bacterial infection. 

### 3.12. Pharmacokinetic Studies 

Based on the data obtained from the physiochemical and antimicrobial evaluations of the prepared CPX ophthalmic formulations, formulation A3 was selected for in vivo pharmacokinetic experiment along with the commercial CPX eye drops, Ciloxan^®^. Fifty μL dose of CPX ophthalmic formulation was applied in the lower conjunctival sac of the rabbit’s right eye; however, Ciloxan^®^ (Riyadh Pharma, Riyadh, Saudi Arabia) was applied in the left one. The animals were euthanized after withdrawing the aqueous humor at specific time intervals. The samples were centrifuged for 20 min at 4 °C and 20,000 rpm and supernatant were frozen at –20 °C until assay. The amount of CPX in each sample was analyzed using the HPLC method described above [27].

### 3.13. Pharmacokinetic Parameters

The C_max_ and T_max_ values were obtained directly from rabbit aqueous humor concentration-time plot. The AUC_0–t_ (μg min/mL) was calculated using the trapezoidal method. The elimination rate constant (k) was determined from the final segment of the curve by fitting the data in first order elimination graph. Relative bioavailability of CPX-CLBF was obtained in comparison to that of the commercial product. 

### 3.14. Statistical Analysis

All experiments in this study were subjected to one way analysis of variance with Tukey’s multiple comparisons test at a 5% level of significance (*p* ≤ 0.05).

## 4. Conclusions

The MIC for CPX lipid-based formulations was noticeably lower than that of the retailed and free drug, indicating that CLBF systems may participate in the increased antimicrobial activity of CPX. The pharmacokinetic parameters of the selected ophthalmic formulation showed significant improvement in ocular bioavailability of CPX in comparison with Ciloxan^®^ ophthalmic drops. This could be due to several factors that characterize the tested CPX formulation, including that it produces low lacrimation due to its almost neutral pH value of 7.4. In addition, the presence of a positive charge inducing agent which increases the drug contact time with corneal surface. Finally, the liposomal formulation improves drug permeability to the cornea which maximizes drug concentration in the affected area. Based on these results, the prepared CPX formulation could provide a better alternative to the marketed one with respect to enhancing ocular drug activity, especially in the management of post-surgical infections.

## Figures and Tables

**Table 1:** Antibacterial activity of selected ophthalmic ciprofloxacin (CPX) colloidal lipid-based formulations (CPX-CLBFs) and the commercial one.

	Gram-Positive Bacteria					
**Formulation Code**	*S. aureus* ATCC 29213		*B. subtilis* ATCC 10400		*E. faecalis* ATCC 29212	
	Sensitivity	MIC	Sensitivity	MIC	Sensitivity	MIC
B0	+++	0.5	++++	0.25	+++	0.125
A3	++++	0.25	++++	0.125	++++	0.125
CPX	++++	0.5	++++	0.25	++++	0.125
	**Gram-Negative Bacteria**					
	*E. coli* ATCC 9637		*P. aeruginosa* ATCC 27953		*K. pneumonia* ATCC 10031	
	Sensitivity	MIC	Sensitivity	MIC	Sensitivity	MIC
B0	++++	0.5	+++	1.0	+++	0.5
A3	++++	0.5	++++	0.25	++++	0.25
CPX	++++	0.5	++++	0.5	++++	0.5

**Table 2:** Pharmacokinetics parameters of ophthalmic CPX- CLBF (A3) and commercial one (mean ± SD, *n* = 3.0).

Parameters	A3	Commercial CPX
Dose (µg)	115	150
C_max_ (µg/mL)	4.2 ± 0.1	2.7 ± 0.1
T_max_ (h)	2	2
K (h^−1^)	0.2 ± 0.03	0.4 ± 0.01
t_1/2_ (h)	3.5 ± 0.2	1.7 ± 0.2
AUC _0–t_ (µg.h/mL)	34.9 ± 2.1	12 ± 0.5
Relative bioavailability	2.9	-

C_max_: maximum plasma concentration; T_max_: time to reach C_max_; K: elimination rate constant; t^1/2^: half-life; AUC_0__–t_: area under curve from time 0 to t.

**Table 3:** Composition of ophthalmic CPX-CLBFs in molar ratios.

Formulation Code	Composition (Molar Ratios)			
	PC	DMPC	CL	STA
A0	1	-	-	-
B0	-	1	-	-
A1	1	-	1	2
B1	-	1	1	2
A2	3	-	1	2
B2	-	3	1	2
A3	6	-	1	2
B3	-	6	1	2

PC: phosphatidylcholine; DMPC: dimyrestoyl phosphatidylcholine; CL: cholesterol; STA: stearylamine.

## Data Availability

This study did not report any data.

## Sample Availability

Samples of the CPX compounds are available from the authors.

## References

[1] Hooper D., Wolfson J., Ng E., Swartz M.J.T. (1987). Mechanisms of action of and resistance to ciprofloxacin. Am. J. Med..

[2] Ting D.S.J., Ho C.S., Deshmukh R., Said R.D.G., Dua H.S. (2021). Infectious keratitis: An update on epidemiology, causative microorganisms, risk factors, and antimicrobial resistance. Eye.

[3] Subrizi A., del Amo E.M., Korzhikov-Vlakh V., Tennikova T., Ruponen M., Urtti A. (2019). Design principles of ocular drug delivery systems: Importance of drug payload, release rate, and material properties. Drug Discov. Today.

[4] Mamah C.C., Anyalebechi O.C., Onwubiko S.N., Okoloagu M.N., Maduka-Okafor F.C., Ebede S.O., Umeh R.E. (2021). Conjunctival bacterial flora and their antibiotic sensitivity among patients scheduled for cataract surgery in a tertiary hospital in south-east Nigeria. Graefes Arch. Clin. Exp. Ophthalmol..

[5] Bhattacharyya A., Sarma P., Sarma B., Kumar S., Gogoi T., Kaur H., Prajapat M. (2020). Bacteriological pattern and their correlation with complications in culture positive cases of acute bacterial conjunctivitis in a tertiary care hospital of upper Assam: A cross sectional study. Medicine.

[6] Jackson T.L., Eykyn S.J., Graham E.M., Stanford M.R. (2003). Endogenous bacterial endophthalmitis: A 17-year prospective series and review of 267 reported cases. Surv. Ophthalmol..

[7] Gieling E.M., Wallenburg E., Frenzel T., Dylan W.L., Schouten J.A., Oever J., Kolwijck E., Burger D.M., Pickkers P., Heine R. (2020). Higher dosage of ciprofloxacin necessary in critically ill patients: A new dosing algorithm based on renal function and pathogen susceptibility. Clin. Pharmacol. Ther..

[8] Mundada A.S., Shrikhande B.K. (2008). Formulation and evaluation of ciprofloxacin hydrochloride soluble ocular drug insert. Curr. Eye Res..

[9] Ludwig A. (2005). The use of mucoadhesive polymers in ocular drug delivery. Adv. Drug Deliv. Rev..

[10] Atugoda T., Wijesekara H., Werellagama D.R.I.B., Jinadasa K.B.S.N., Bolan N.S., Vithanage M. (2020). Adsorptive interaction of antibiotic ciprofloxacin on polyethylene microplastics: Implications for vector transport in water. Environ. Technol. Innov..

[11] Wilhelmus K.R., Abshire R.L. (2003). Corneal ciprofloxacin precipitation during bacterial keratitis. Am. Ophthalmol..

[12] Law S.L., Huang K.J., Chiang C.H. (2000). Acyclovir-containing liposomes for potential ocular delivery: Corneal penetration and absorption. J. Control. Rel..

[13] Lila A.S.A., Ishida T. (2017). Liposomal delivery systems: Design optimization and current applications. Biol. Pharm. Bull..

[14] Danion A., Arsenault I., Vermette P. (2007). Antibacterial activity of contact lenses bearing surface-immobilized layers of intact liposomes loaded with levofloxacin. J. Pharm. Sci..

[15] Perween N., Alshehri S., Easwari T.S., Verma V., Faiyazuddin M., Alanazi A., Shakeel F. (2021). Investigating the feasibility of mefenamic acid nanosuspension for pediatric delivery: Preparation, characterization, and role of excipients. Processes.

[16] Gulati M., Grover M., Singh M., Singh S. (1998). Study of azathioprine encapsulation into liposomes. J. Microencapsul..

[17] Keynan Y., Finkelman Y., Lagacé-Wiens P. (2012). The microbiology of endophthalmitis: Global trends and a local perspective. Eur. J. Clin. Microbiol. Infect. Dis..

[18] Donnenfeld E.D., Perry H.D., Snyder R.W., Elsky M., Jones H. (1997). Intercorneal, aqueous Humor, and vitreous humor penetration of topical and oral ofloxacin. Arch. Ophthalmol..

[19] Leigue L., Montiani-Ferreira F., Moore B.A. (2016). Antimicrobial susceptibility and minimal inhibitory concentration of *Pseudomonas aeruginosa* isolated from septic ocular surface disease in different animal species. Open Vet. J..

[20] Cutarelli P.E., Lass J.H., Lazarus H.M., Putman S.C., Jacobs M.R. (1991). Topical fluoroquinolones: Antimicrobial activity and in vitro corneal epithelial toxicity. Curr. Eye Res..

[21] Lesk M.R., Ammann H., Marcil G., Vinet B., Lamer L., Sebag M. (1993). The penetration of oral ciprofloxacin into the aqueous humor, vitreous, and subretinal fluid of humans. Am. J. Ophthalmol..

[22] Jain R.L., Shastri J.P. (2011). Study of ocular drug delivery system using drug-loaded liposomes. Int. J. Pharm. Investig..

[23] Szoka F., Papahadjopoulos D. (1978). Procedure for preparation of liposomes with large internal aqueous space and high capture by reverse-phase evaporation. Proc. Nat. Acad. Sci. USA.

[24] Gürsoy A., Senyücel B. (1997). Characterization of ciprofloxacin liposomes: Derivative ultraviolet spectrophotometric determinations. J. Microencapsul..

[25] Akanksha G., Navneet G., Vivek T., Mayank M. (2007). Topical liposomal gel with aceclofenac: Characterization and in vitro evaluation. Pharmacist.

[26] Budai L., Hajdú M., Budai M., Gróf P., Béni S., Noszál B., Klebovich I., Antal I. (2007). Gels and liposomes in optimized ocular drug delivery: Studies on ciprofloxacin formulations. Int. J. Pharm..

[27] Zotou A., Miltiadou N. (2002). Sensitive LC determination of ciprofloxacin in pharmaceutical preparations and biological fluids with fluorescence detection. J. Pharm. Biomed. Anal..

[28] Paulsson M., Hägerström H., Edsman K. (1999). Rheological studies of the gelation of deacetylated gellan gum (Gelrite) in physiological conditions. Eur. J. Pharm. Sci..

[29] Avgoustakis K., Beletsi A., Panagi Z., Klepetsanis P., Karydas A.G., Ithakissios D.S. (2002). PLGA-mPEG nanoparticles of cisplatin: In vitro nanoparticle degradation, in vitro drug release and in vivo drug residence in blood properties. J. Control Rel..

[30] Balouiri M., Sadiki M., Ibnsouda S.K. (2016). Methods for in vitro evaluating antimicrobial activity: A review. J. Pharm. Anal..

[31] Wiegand I., Hilpert K., Hancock R.E.W. (2008). Agar and broth dilution methods to determine the minimal inhibitory concentration (MIC) of antimicrobial substances. Nat. Protoc..

[32] CLSI WL: M02-A11 (2015). Performance Standards for Antimicrobial Disk Susceptibility Tests; Approved Standard.

[33] Callegan M.C., Gilmore M.S., Gregory M., Ramadan R.T., Wiskur B.J., Moyer A.L., Hunt J.J., Novosad B.D. (2007). Bacterial endophthalmitis: Therapeutic challenges and host–pathogen interactions. Prog. Retin. Eye Res..

[34] Otvos L., Cudic M. (2007). Broth microdilution antibacterial assay of peptides. Methods Mol. Biol..

[35] EUCAST SOP 1.1 (2013). Testing ECoAS: Setting Breakpoints for New Antimicrobial Agents.

